# AI in motion: the impact of data augmentation strategies on mitigating MRI motion artifacts

**DOI:** 10.1007/s00330-025-11670-6

**Published:** 2025-05-17

**Authors:** Simon D. Westfechtel, Kristoffer Kußmann, Cederic Aßmann, Marc S. Huppertz, Robert M. Siepmann, Teresa Lemainque, Vera R. Winter, Alexandra Barabasch, Christiane K. Kuhl, Daniel Truhn, Sven Nebelung

**Affiliations:** Department for Diagnostic and Interventional Radiology, University Hospital Aachen, Aachen, Germany

**Keywords:** Lower limbs, Magnetic resonance imaging, Artificial intelligence, Torsion abnormality, Artifacts

## Abstract

**Objectives:**

Artifacts in clinical MRI can compromise the performance of AI models. This study evaluates how different data augmentation strategies affect an AI model’s segmentation performance under variable artifact severity.

**Materials and methods:**

We used an AI model based on the nnU-Net architecture to automatically quantify lower limb alignment using axial T2-weighted MR images. Three versions of the AI model were trained with different augmentation strategies: (1) no augmentation (“baseline”), (2) standard nnU-net augmentations (“default”), and (3) “default” plus augmentations that emulate MR artifacts (“MRI-specific”). Model performance was tested on 600 MR image stacks (right and left; hip, knee, and ankle) from 20 healthy participants (mean age, 23 ± 3 years, 17 men), each imaged five times under standardized motion to induce artifacts. Two radiologists graded each stack’s artifact severity as none, mild, moderate, and severe, and manually measured torsional angles. Segmentation quality was assessed using the Dice similarity coefficient (DSC), while torsional angles were compared between manual and automatic measurements using mean absolute deviation (MAD), intraclass correlation coefficient (ICC), and Pearson’s correlation coefficient (r). Statistical analysis included parametric tests and a Linear Mixed-Effects Model.

**Results:**

MRI-specific augmentation resulted in slightly (yet not significantly) better performance than the default strategy. Segmentation quality decreased with increasing artifact severity, which was partially mitigated by default and MRI-specific augmentations (e.g., severe artifacts, proximal femur: DSC_baseline_ = 0.58 ± 0.22; DSC_default_ = 0.72 ± 0.22; DSC_MRI-specific_ = 0.79 ± 0.14 [*p* < 0.001]). These augmentations also maintained precise torsional angle measurements (e.g., severe artifacts, femoral torsion: MAD_baseline_ = 20.6 ± 23.5°; MAD_default_ = 7.0 ± 13.0°; MAD_MRI-specific_ = 5.7 ± 9.5° [*p* < 0.001]; ICC_baseline_ = −0.10 [*p* = 0.63; 95% CI: −0.61 to 0.47]; ICC_default_ = 0.38 [*p* = 0.08; −0.17 to 0.76]; ICC_MRI-specific_ = 0.86 [*p* < 0.001; 0.62 to 0.95]; r_baseline_ = 0.58 [*p* < 0.001; 0.44 to 0.69]; r_default_ = 0.68 [*p* < 0.001; 0.56 to 0.77]; r_MRI-specific_ = 0.86 [*p* < 0.001; 0.81 to 0.9]).

**Conclusion:**

Motion artifacts negatively impact AI models, but general-purpose augmentations enhance robustness effectively. MRI-specific augmentations offer minimal additional benefit.

**Key Points:**

***Question*** *Motion artifacts negatively impact the performance of diagnostic AI models for MRI, but mitigation methods remain largely unexplored.*

***Findings*** *Domain-specific augmentation during training can improve the robustness and performance of a model for quantifying lower limb alignment in the presence of severe artifacts.*

***Clinical relevance*** *Excellent robustness and accuracy are crucial for deploying diagnostic AI models in clinical practice. Including domain knowledge in model training can benefit clinical adoption.*

**Graphical Abstract:**

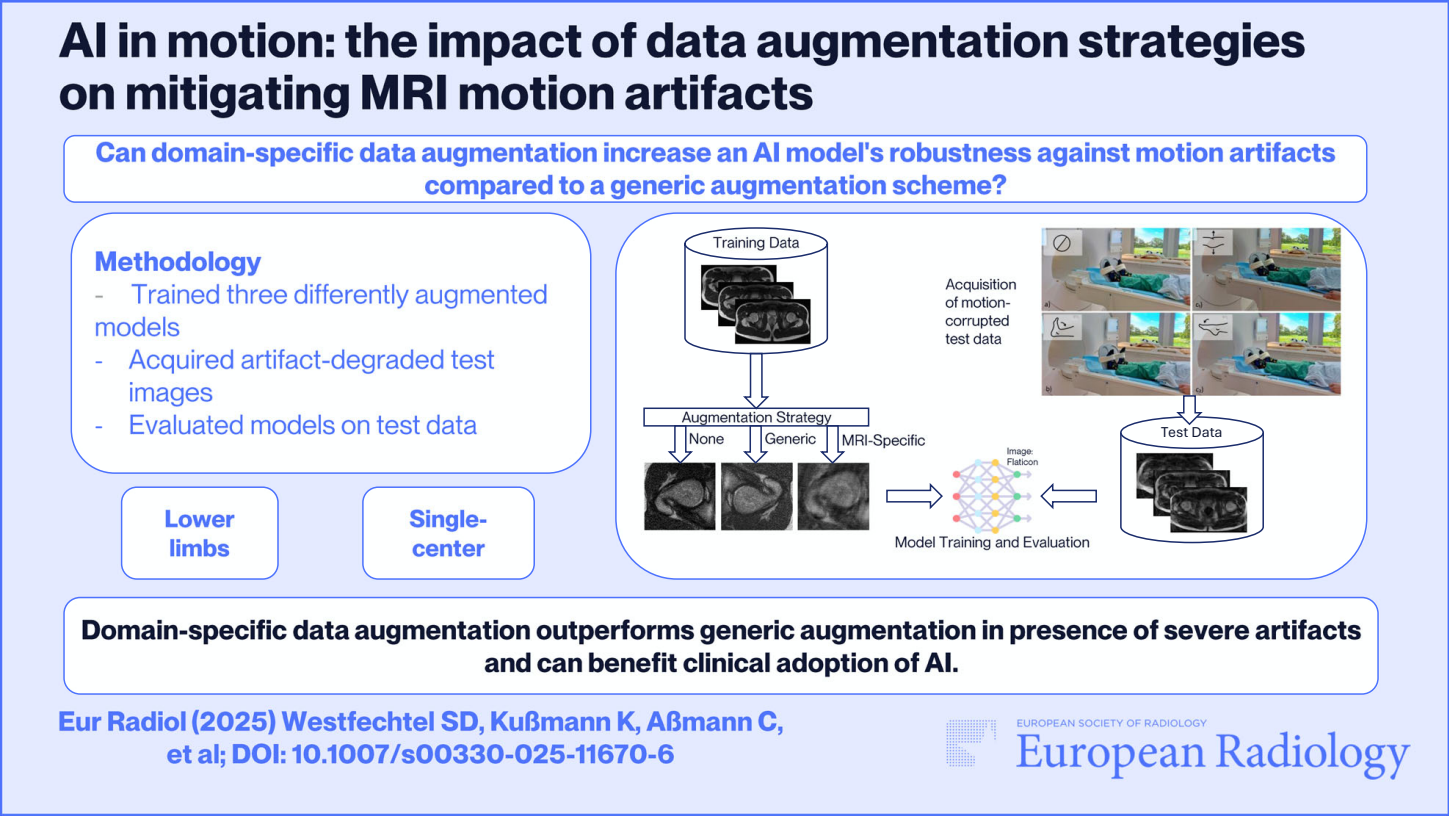

## Introduction

Automated image processing has become a focus of scientific and clinical attention in radiology and beyond [1]. Modern computer vision methods may aid clinicians in their work by automating time-intensive and repetitive tasks. Diagnostic artificial intelligence (AI) models are often developed under well-controlled (“laboratory”) conditions, where the training data is carefully curated, the image quality is consistent, and variability regarding image contrast, resolution, noise, patient position, and artifacts is low. Although widely perceived as the ultimate imaging modality of contemporaneous clinical medicine [2] due to its excellent soft tissue contrast and absence of ionizing radiation, MRI is a complex technology susceptible to artifacts [3]. Artifact-ridden imaging studies can seriously hamper diagnostic accuracy and make images non-diagnostic [4]. One of the most common artifact types is motion artifacts secondary to (in)voluntary patient motion during image acquisition, which present as blurring, ghosting, or smearing of image parts or entire images in the phase-encoding direction. Motion artifacts affect up to a third of clinical MRI sequences, and approximately 20% of MRI studies require repetition of a scan due to motion corruption of the image, which increases scan times, reduces patient comfort, and causes additional costs [5, 6]. Artifact-induced image degradation also negatively affects the performance of diagnostic AI models. Consequently, considerable effort has been directed at reducing artifacts prospectively during image acquisition, for example, by employing radial sampling (commonly known by proprietary sequence names PROPELLER, BLADE, or MULTIVANE). In some instances, motion artifacts are present despite using prospective motion correction, while in others, these techniques are not available or feasible. Thus, other efforts focused on artifact mitigation during image reconstruction using deep learning models to remove artifacts from degraded images, for example, by utilizing generative adversarial networks [7, 8]. However, this approach has several drawbacks, e.g., poor generalizability to other use cases or increased complexity due to the combination of multiple models. A less explored alternative focuses on improving diagnostic AI models’ robustness when dealing with artifacts during image analysis, for example, by using data augmentation techniques to enhance the training data in volume, quality, and diversity.

In musculoskeletal imaging, AI models are used for various tasks, including automatically segmenting anatomic structures such as bones [9]. Segmentation models are frequently combined with computational post-processing techniques [10], such as quantification of lower limb alignment [11–13], which is critical for clinical management. Aberrant femoral and tibial alignment is associated with numerous pathologies and symptoms, including pain and dysfunction, femoroacetabular impingement, and hip osteoarthritis [14, 15]. In the clinic, the physician manually identifies specific anatomic landmarks on cross-sectional images, constructs reference lines connecting these landmarks, and quantifies the torsional angle [16, 17]. Due to substantial intra- and inter-reader variability, ranging up to 11° and 16° [18], and challenges associated with inconsistent measurement levels and methods [16, 19], scientific efforts have been directed at automating the quantification procedure [11–13]. Nonetheless, little is known about how diagnostic AI models are impacted by motion artifacts or how data augmentation strategies, i.e., using different techniques to enhance the training data’s volume, quality, and diversity, may mitigate these artifacts to increase clinical robustness. Our objective was to systematically study the accuracy of lower limb segmentation and quantification as a function of artifact severity and data augmentation strategy. We hypothesized that a dedicated AI model’s performance would decrease with increasing artifact severity but could be stabilized by domain-specific data augmentation during training.

## Materials and methods

### Study design

Following approval by the local Ethics Committee (Ethics Committee of the medical faculty of RWTH Aachen University, EK 058/22), informed consent from the test set participants was obtained in written form, while this requirement was waived for the training set patients. This study was designed as a comparative evaluation of the impact of three different data augmentation strategies on the performance of an AI model that automatically segments the lower limbs and quantifies their alignment. We used image data from a previous study [13] for the training and validation sets and prospectively acquired data for the test set.

### Training and validation sets

**Fig. 1:**
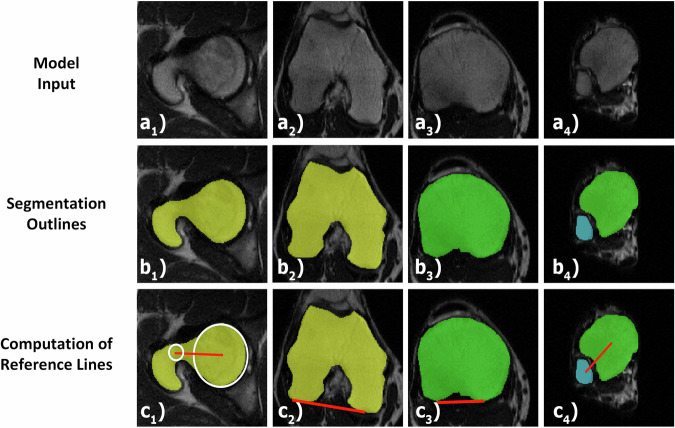
Diagnostic AI model for quantifying lower limb torsion. The model inputs are axial MR images of the hips (**a**_**1**_), knees (**a**_**2**_, **a**_**3**_), and ankles (**a**_**4**_) (only the patient’s right side is shown). Using a convolutional neural network, the model outputs segmentation outlines of the femur (yellow; **b**_**1**_, **b**_**2**_), tibia (green; **b**_**3**_, **b**_**4**_), and fibula (blue; **b**_**4**_). Algorithmic post-processing then identifies anatomic landmarks based on these segmentation outlines and defines reference lines (red) according to the method by Lee et al (**c**_**1**_, **c**_**2**_) and the ellipses method (**c**_**3**_, **c**_**4**_). Femoral and tibial torsion are then quantified based on these reference lines. White circles indicate accessory geometric structures to identify the centers of the femoral head and neck (**c**_**1**_)

This study builds upon previous work by Schock et al, where we developed and validated an AI model to measure lower limb alignment [13]. The present study used the identical axial T2-weighted non-fat-saturated 2D turbo-spin echo sequences—the clinical standard sequence for the MR-based assessment of torsional alignment [20]—acquired on a clinical 3.0-T MRI scanner (Achieva, Philips). The scans came from the clinical routine and included bilateral stacks of the hips, knees, and ankles of 93 patients (mean age, 13 ± 5 years; 52 males; for date range and in/exclusion criteria, refer to the original publication). Along with the MRI data, expert-checked manual segmentation outlines of the femur, tibia, and fibula were available. A U-net convolutional neural network was trained to segment these bones, followed by a post hoc algorithmic identification of anatomic landmarks, definition of reference lines, and quantification of torsional alignment (Fig. 1). The patients were allocated to a training (*n* = 74) and validation set (*n* = 19). Altogether, 186 lower limbs, each consisting of three joint-level image stacks and manual segmentation outlines of the proximal and distal femur, the proximal and distal tibia, and the distal fibula, were available for training and validation.

### Test set

**Fig. 2:**
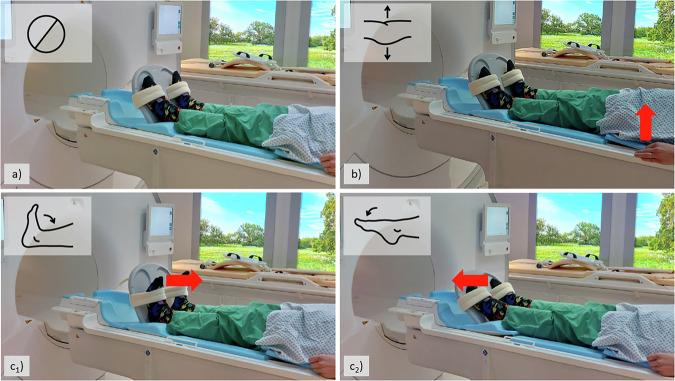
Standardized motion patterns for generating motion artifact-degraded MR images for the test set. Five axial T2-weighted non-fat-saturated 2D turbo-spin echo sequences were acquired consecutively under different conditions: with participants lying as still as possible (**a**), performing breath-synchronized repetitive (unilateral, yet alternating) gluteal contractions and relaxations (**b**) and breath-synchronized repetitive dorsiflexion (**c**_**1**_) and plantarflexion (**c**_**2**_). Red arrows indicate the direction of motion

The test set was compiled by prospectively acquiring MRI studies of 20 healthy participants (aged 23 ± 3 years, 17 men, acquired between 04/2023 and 09/2023) on a clinical 3.0-T MRI scanner (Elition X, Philips). The participants were imaged supine with extended knees and patellae facing anteriorly, supported by a dedicated flexible footrest. Axial T2-weighted non-fat-saturated 2D turbo-spin echo sequences were acquired over the hips, knees, and feet in separate stacks, using the inbuilt body coil and without repositioning (refer to Supplementary Text for sequence details). This sequence was acquired five times per participant as follows (Fig. 2):

- In the resting position with participants as still as possible ([i] reference),
- Under breath-synchronized repetitive foot motion (from maximum dorsiflexion during inspiration to maximum plantarflexion during expiration) at high frequency (one complete motion amplitude completed with every breath cycle [ii]) or at low frequency (with every third breath cycle [iii]),
- Under breath-synchronized repetitive maximum tensioning of the gluteal muscles, i.e., gluteal contraction during inspiration and relaxation during expiration and alternating between right and left, at high frequency [iv] or low frequency [v].

**Fig. 3:**
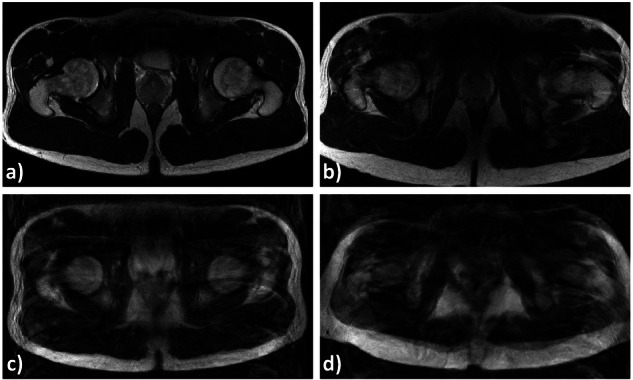
Representative MR images showing motion artifact-induced image degradation. Axial T2-weighted non-fat-saturated images of the pelvis, displaying both hips in different participants, are shown. The images were evaluated for motion artifact-induced degradation and categorized as showing no (**a**), mild (**b**), moderate (**c**), and severe (**d**) degradation

Following the acquisition of these five series per participant, i.e., 100 series in total, the axial stacks of each joint level, i.e., 600 image stacks in total, were evaluated by two clinical radiologists (M.S.H. and R.M.S., with 4 years of experience in musculoskeletal MRI) who assessed the severity of artifact-induced image degradation using the method of Kohli et al [21]. Each stack was classified as displaying no, mild, moderate, and severe motion artifacts (Fig. 3) independently by each reader. Image stacks with differing artifact severity scores were discussed until a consensus score was agreed on. The clinical radiologists also compared in silico augmented MR images with artifact-degraded MR images from the test set, rating the in-plane and through-plane similarity using a Likert scale (1 [no similarity] to 5 [maximum similarity]).

An à-priori sample size estimation that was performed using G*Power (v3.1.9.7, F tests, repeated-measures ANOVA within factors, f = 0.25 [medium effect size; prior research suggests limited clinical relevance], α = 0.05, power = 0.80, corr = 0.6 [correlation among repeated measures]) indicated that approximately 17 participants would be needed.

### Model description

**Fig. 4:**
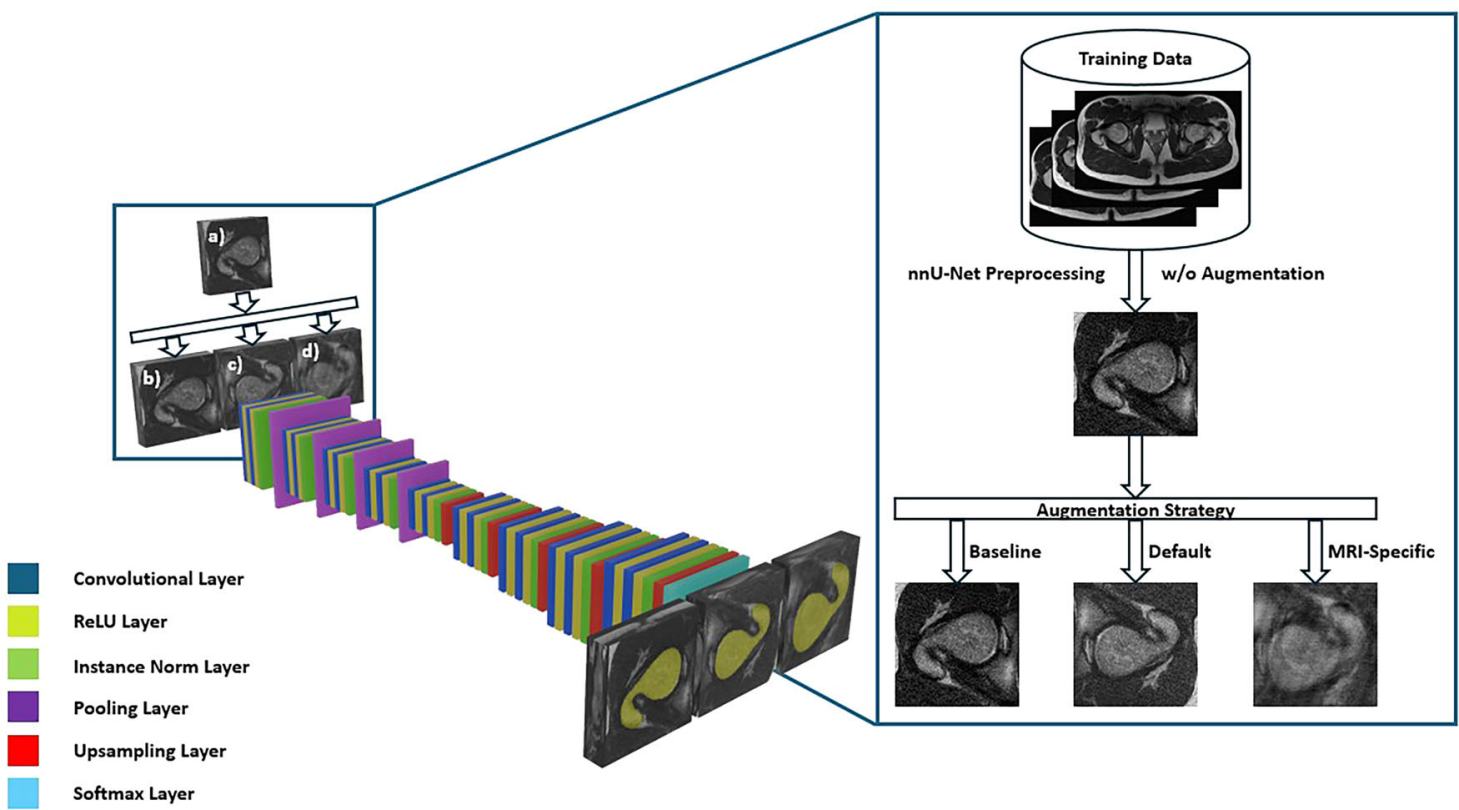
Schematic of neural network architecture and model pipeline for automatic bone segmentation using different augmentation strategies. During training, the original MR images (**a**) were either left unchanged (**b**, “baseline”), augmented with default nnU-Net augmentations (**c**, “default”), or augmented with additional MRI-specific augmentation (**d**, “MRI-specific”). The neural network’s topological characteristics to delineate the femoral segmentation outlines (yellow) are shown. For illustrative purposes, “default” augmentations are shown as mirroring and contrast transformations, while “MRI-specific” augmentations include additional random motion, ghosting, and spiking. The blue box on the right details the steps not visualized in the blue box on the left. Here, “nnU-Net Preprocessing w/o Augmentation” refers to the standard preprocessing steps applied by nnU-Net, such as resampling, normalization, and cropping/padding, while excluding any data augmentation

An updated AI model based on the one from Schock et al [13] was trained using three different augmentation strategies during training, resulting in three versions that used the same model architecture. Figure 4 illustrates the network architecture and the modified training pipeline.

#### Preprocessing including data augmentation

Preprocessing was performed using functionality provided by the nnU-Net Python package (version 2.3.1). Briefly, images were cropped to their non-zero regions, normalized using z-scoring, and resampled to a common shape and voxel spacing using nearest neighbor interpolation due to the anisotropic nature of the images. Parameters for these preprocessing steps were dynamically derived from a dataset fingerprint; therefore, no fixed values were specified. The target voxel spacing was determined by analyzing the dataset’s voxel spacings and computing their statistics, as described for the nnU-net methodology [22]. Three model versions with different augmentation strategies were implemented:

1. **“Baseline”**: Without augmentations.
2. **“Default”**: With default nnU-Net augmentations [22, 23], including rotation, rescaling, mirroring, Gaussian noise addition, brightness/contrast changes, resolution reduction, and gamma adjustments.
3. **“MRI-specific”**: With default nnU-Net augmentations plus MRI-specific augmentations, including random blurring, Herringbone-artifact-like stripes (spiking), signal intensity variations, structural rotation/translation (due to patient motion), and ghosting artifacts, i.e., displaced and superimposed duplicate structures in the image [24]. These artifacts were modeled in silico using the Torchio Python library [25] and the parameters listed in Supplementary Table 1. Each artifact was employed with a probability of 50%, except for spikes (probability of 2%). Exemplary images of these augmentations are presented in Supplementary Fig. 1.

#### Segmentation model

Reflecting current developments in the field, we updated the earlier AI model from Schock et al [13] as follows. First, we incorporated the state-of-the-art 3D nnU-Net architecture (version 2.3.1) [22], replacing the conventional U-net architecture [26]. The nnU-Net builds on the well-studied U-Net convolutional neural network but automates various aspects of model configuration and training [22]. It uses an encoder-decoder architecture to enable precise localization while adapting to different datasets without requiring manual tuning. Additional adjustments include limiting the batch size to allow for larger patch sizes, replacing batch normalization with instance normalization, and using leaky linear units instead of standard rectified ones. Individual models use a common template that is automatically configured based on the properties of the training data. Specifically, we used an adapted trainer class, implementing TorchIO transforms for data augmentation and default hyperparameters for model training, except for an increased number of epochs (*n* = 500 from an original *n* = 150 [in version 2.3.1]). This increase was necessary to handle the increased complexity introduced by additional augmentations. The final model was selected based on the validation set’s highest Dice Similarity Coefficient (DSC).

#### Post-processing

Automated quantification of torsional alignment followed the implementation of Schock et al [13]. Based on computed segmentation outlines, reference lines and angles were calculated using an algorithmic approach. Briefly, the algorithm automated the Lee method [27] for calculating femoral torsion and the Ulm method [28] for tibial torsion. A least-squares fit was used to identify the centers of the femoral head and neck. In contrast, the posterior femoral and tibial condyles (of the knee) were identified by iteratively shifting a line in the anterior direction, and the centers of the distal tibia and fibula were identified by computing the centroids of the respective segmentation outlines. Supplementary Table 2 provides further details on the definition of the reference lines. The original publication by Schock et al provides an in-depth explanation of the algorithm. The code is available at https://github.com/swestfechtel/paper-augmentation.

### Evaluation of model performance

The impact of the augmentation strategies during training was assessed on the test set. Since nnU-Net prioritizes the DSC as its evaluation metric, we measured segmentation quality using the DSC for each bone—the femur, tibia, and fibula—in every image stack. This metric quantifies the similarity between manual and automatic segmentation outlines as a measure of segmentation quality. Manual reference segmentation outlines were produced by a pre-graduate medical student trained by an experienced musculoskeletal radiologist (S.N. with ten years of experience) on the resting-position series. Quality and consistency checks were performed by M.S.H., R.M.S., and S.N.

During axial MR image acquisition, repetitive gluteal contraction and relaxation primarily caused in-plane motion artifacts, typically manifesting as blurring, ghosting, or streaking. In contrast, repetitive foot dorsiflexion and plantarflexion caused additional through-plane motion artifacts, manifesting as misaligned and discontinuous anatomy between slices. Consequently, image stacks of the feet graded as compromised by motion artifacts were registered to their corresponding reference stacks using a 2D similarity transform [29], determined via regular step gradient descent to maximize mutual information between images.

M.S.H. and R.M.S. independently determined femoral and tibial torsion on the test data using standard angle measurement functionalities provided in the in-house PACS (Philips, IntelliSpace, v4.4553.35).

### Statistical analysis

The statistical analysis was performed by S.D.W. using Python (v3.11.5) and the pandas (v2.1.4) library and R (v4.4.0) with its packages afex (v1.3-1) and emmeans (v1.10.1). A linear mixed-effects model was fit to quantify the coefficients (and *p*-values) for each contributing variable: participant (random), joint level (fixed), and artifact severity (fixed), and their effects on segmentation quality based on augmentation strategy. Coefficients are given as means with 95% confidence intervals.

For each joint level and artifact severity, repeated-measures ANOVA was used to assess whether DSC values differed significantly between the augmentation strategies, following normality testing using the Shapiro–Wilk test. Post hoc pairwise comparisons were conducted using Tukey’s Honest Significant Difference test.

The significance level was set to *p* ≤ 0.05 and further stratified as 0.01 < *p* ≤ 0.05 (*), 0.001 < *p* ≤ 0.01 (**), and *p* ≤ 0.001 (***). The inter-rater reliability of the two radiologists’ manual measurements was evaluated using the Intraclass Correlation Coefficient (ICC). Furthermore, repeated-measures ANOVA of Mean Absolute Deviation (MAD), the ICC, and Pearson’s Correlation Coefficient r were computed to compare automated and manual torsion measurements and used as an additional gauge for model performance.

## Results

### Study sample

Ninety-three patients (mean age 13 ± 5 years, 52 male) were included in the training and validation sets, and 20 participants (23 ± 3 years, 17 male) in the test sets. Artifact severity was close to equally distributed in the test set (Supplementary Table 3).

### Augmentation quality

While the in-plane similarity between in silico augmented and real MR image datasets was consistently high across all joints and artifact severities (mild artifacts: 3.6 ± 0.6; moderate artifacts: 3.6 ± 0.5; severe artifacts: 3.8 ± 0.2), the through-plane similarity was only modest (mild artifacts: 2.6 ± 0.5; moderate artifacts: 2.7 ± 0.6; severe artifacts: 3.1 ± 0.5). In real MR image stacks, motion-induced artifacts varied in location and intensity between slices, while the in silico-generated artifacts were constant across all slices, leading to reduced through-plane correspondence.

### Segmentation quality

**Fig. 5:**
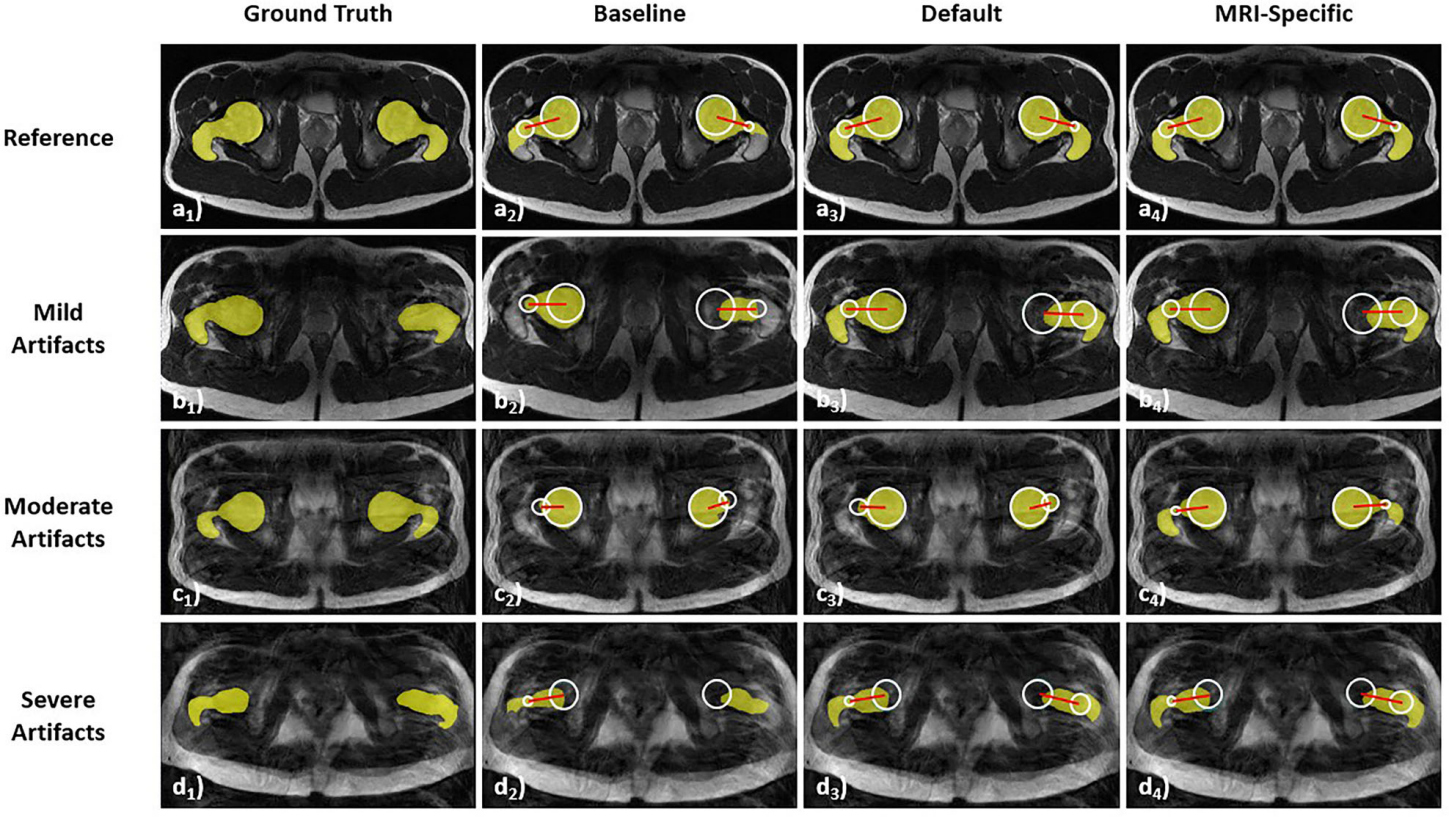
Segmentation quality as a function of augmentation strategy and artifact severity. MR images with overlaid segmentation outlines of the hip are shown. The reference image is unaffected by artifacts, while the other images are affected by varying degrees of artifact severity, from mild to severe. Color coding and image overlays as in Fig. 1. Absence of reference lines (**d**_**2**_) indicates that no reference line could be computed. Improved segmentation and post hoc processing were observed with more extensive augmentation during training when fewer artifacts were present. For these images, the Dice Similarity Coefficients (DSC) were as follows. Reference image: 0.91 (**a**_**2**_), 0.96 (**a**_**3**_), 0.96 (**a**_**4**_). Image with mild artifacts: 0.58 (**b**_**2**_), 0.85 (**b**_**3**_), 0.84 (**b**_**4**_). Image with moderate artifacts: 0.51 (**c**_**2**_), 0.65 (**c**_**3**_), 0.82 (**c**_**4**_). Image with severe artifacts: 0.84 (**d**_**2**_), 0.89 (**d**_**3**_), 0.89 (**d**_**4**_). Manual and computed femoral torsional values (R/L [°]) were as follows. Reference image: 9.5/12.4 (**a**_**1**_), 12.9/12.7 (**a**_**2**_), 12.7/12.0 (**a**_**3**_), 10.6/11.8 (**a**_**4**_). Image with mild artifacts: 6.0/−0.3 (**b**_**1**_), 5.1/−0.5 (**b**_**2**_), 5.3/−0.4 (**b**_**3**_), 5.7/−0.3 (**b**_**4**_). Image with moderate artifacts: −0.9/−1.5 (**c**_**1**_), −0.1/−6.0 (**c**_**2**_), −0.1/−5.5 (**c**_**3**_), −0.9/−1.2 (**c**_**4**_). Image with severe artifacts: 10.3/12.2 (**d**_**1**_), 11.5/NA (**d**_**2**_), 11.5/16.0 (**d**_**3**_), 11.5/13.6 (**d**_**4**_)

Segmentation quality was quantified for each joint level and bone. It was affected by artifact severity, anatomic region, and augmentation strategy (Table 1, Fig. 5). The associated post hoc test results are detailed in Supplementary Tables 4–8.

**Table 1:** Quantification of segmentation quality (dice similarity coefficients) as a function of artifact severity, augmentation strategy, and anatomic region

		Anatomic region				
Artifact severity	Augmentation strategy	Proximal femur	Distal femur	Proximal tibia	Distal tibia	Distal fibula
Reference	Baseline	0.92 ± 0.03	0.78 ± 0.05	0.33 ± 0.23	0.63 ± 0.15	0.70 ± 0.17
	Default	0.96 ± 0.01	0.97 ± 0.01	0.97 ± 0.01	0.92 ± 0.08	0.83 ± 0.19
	MRI-specific	0.96 ± 0.01	0.97 ± 0.01	0.97 ± 0.01	0.92 ± 0.09	0.83 ± 0.19
	*p*-value	ns	***	***	***	***
Mild	Baseline	0.86 ± 0.05	0.70 ± 0.06	0.30 ± 0.20	0.52 ± 0.23	0.59 ± 0.24
	Default	0.89 ± 0.05	0.87 ± 0.07	0.86 ± 0.08	0.82 ± 0.14	0.69 ± 0.21
	MRI-specific	0.89 ± 0.05	0.87 ± 0.07	0.86 ± 0.08	0.82 ± 0.14	0.69 ± 0.21
	*p*-value	ns	***	***	***	***
Moderate	Baseline	0.83 ± 0.07	0.71 ± 0.06	0.33 ± 0.21	0.39 ± 0.21	0.49 ± 0.23
	Default	0.88 ± 0.05	0.86 ± 0.06	0.85 ± 0.07	0.82 ± 0.11	0.70 ± 0.16
	MRI-specific	0.88 ± 0.05	0.86 ± 0.07	0.85 ± 0.08	0.82 ± 0.11	0.71 ± 0.16
	*p*-value	ns	***	***	***	***
Severe	Baseline	0.58 ± 0.22	0.71 ± 0.08	0.42 ± 0.28	0.13 ± 0.16	0.08 ± 0.17
	Default	0.72 ± 0.22	0.85 ± 0.03	0.82 ± 0.04	0.72 ± 0.20	0.58 ± 0.19
	MRI-specific	0.79 ± 0.14	0.84 ± 0.04	0.82 ± 0.04	0.75 ± 0.12	0.59 ± 0.19
	*p*-value	***	***	***	***	***

Dice Similarity Coefficients are indicated as means ± standard deviation. One-way ANOVA was used to compare the Dice Similarity Coefficients across different augmentation strategies for each anatomic region and artifact severity. The corresponding *p*-values are organized row-wise. *p*-value coding: [***], *p* < 0.001; [ns], non-significant. Details of post hoc pairwise tests are indicated in Supplementary Tables 4–8

On reference images, segmentation quality was excellent for all anatomic regions if augmentation was used during training, with mean DSC values ranging between 0.83 and 0.97. In contrast, the baseline model was characterized by highly variable mean DSC values ranging from 0.33 (proximal tibia) to 0.92 (proximal femur). Increasing artifact severity led to lower DSC values, indicating reduced segmentation accuracy. Augmentation partially mitigated this deterioration in segmentation accuracy. More specifically, although the augmented models demonstrated improved performance compared to the baseline on artifact-degraded images, their segmentation accuracy did not reach the levels achieved on artifact-free images.

The baseline model demonstrated lower DSC values compared to default and MRI-specific augmentations. Around the knee and ankle, these differences were significant for all levels of artifact severity, while at the hip (proximal femur), these differences were significant only for severe artifacts. MRI-specific augmentation showed slight but non-significant improvements in segmentation quality compared to default augmentation for severe artifacts in the proximal femur (0.79 ± 0.14 vs. 0.72 ± 0.22 [*p* = 0.18]) and in the distal tibia (0.75 ± 0.12 vs. 0.71 ± 0.20 [*p* = 0.45]). For other regions, default and MRI-specific augmentations produced similar DSC values.

When quantifying each variable’s contribution to segmentation quality (Table 2), we found that:

(i) Segmentation outlines of the proximal femur had significantly higher DSC values than those of other joint levels, except for the distal femur.
(ii) MR images without artifacts had significantly higher-quality segmentation outlines than those with artifacts.
(iii) Training with default and MRI-specific augmentations improved segmentation quality significantly compared to the baseline model.

**Table 2:** Quantitative evaluation of variables affecting segmentation quality

Variable	Reference	Coefficient	95% confidence interval	*p*-value
Distal femur	Proximal femur	−0.025	[−0.060, 0.010]	0.06
Proximal tibia	Proximal femur	−0.162	[−0.187, −0.137]	**< 0.001**
Distal tibia	Proximal femur	−0.167	[−0.192, −0.142]	**< 0.001**
Distal fibula	Proximal femur	−0.233	[−0.258, −0.208]	**< 0.001**
Mild artifacts	No artifacts	−0.101	[−0.125, −0.077]	**< 0.001**
Moderate artifacts	No artifacts	−0.117	[−0.141, −0.093]	**< 0.001**
Severe artifacts	No artifacts	−0.222	[−0.247, −0.197]	**< 0.001**
Default	Baseline	0.281	[0.261, 0.301]	**< 0.001**
MRI-specific	Baseline	0.287	[0.267, 0.307]	**< 0.001**

The contributions of the different variables to segmentation quality, measured as Dice Similarity Coefficients (DSC), were quantified using a linear mixed-effects model. The table lists variables, references, coefficients, confidence intervals, and *p*-values. Coefficients are reported relative to their reference, i.e., they indicate the relative increase or decrease in DSC values compared to the corresponding reference. For example, a coefficient value of +0.281 for “default augmentation” means that the mean DSC value is 0.281 higher with default augmentation than without augmentation. Significant differences are indicated in bold type

### Torsional angle measurements

Manual torsional angle quantification was a reliable reference standard for inter-method comparisons; the two radiologists demonstrated excellent inter-reader reliability with an ICC of 0.94 (95% CI: 0.93 to 0.95).

**Fig. 6:**
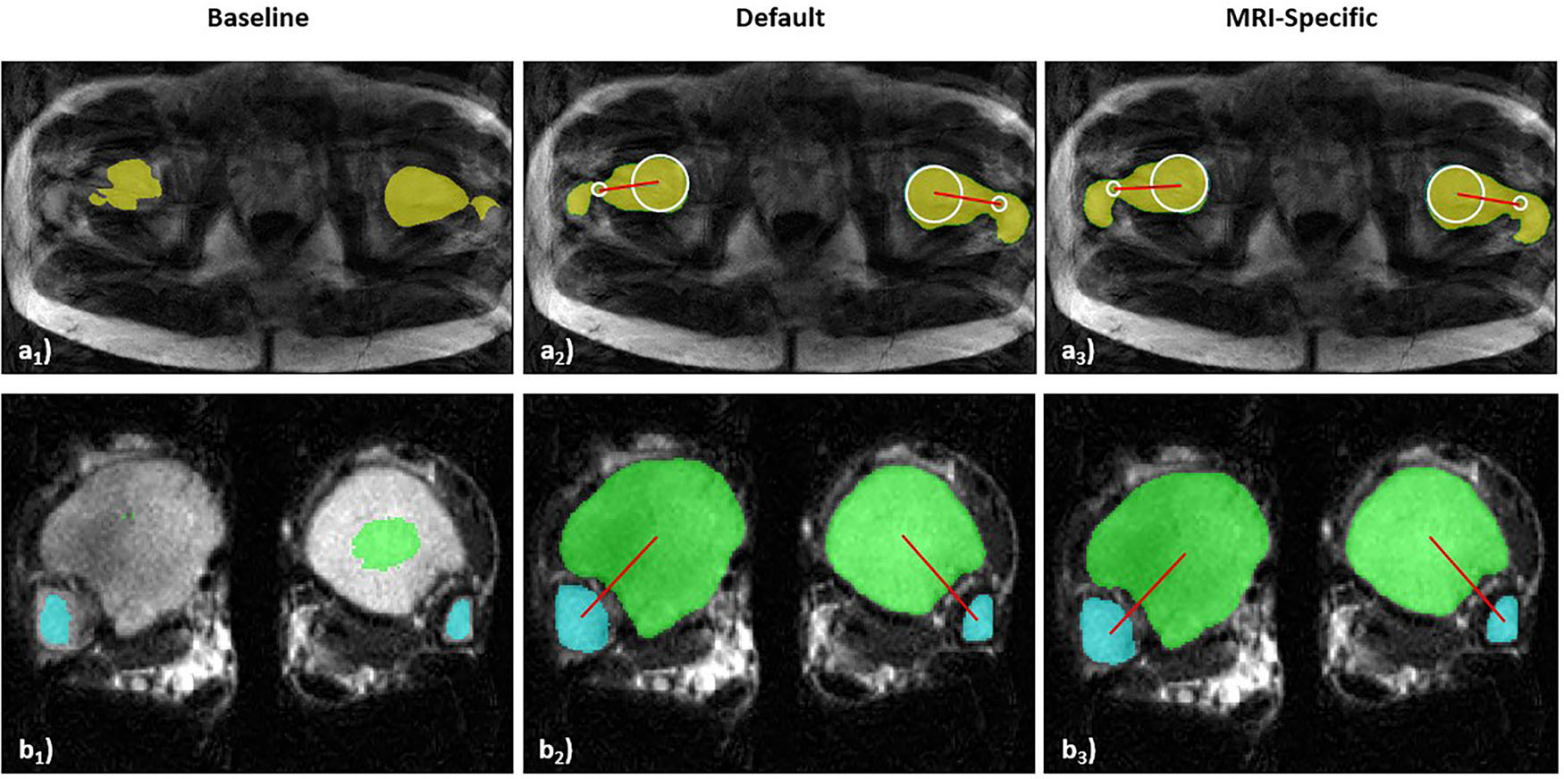
Examples of failed automatic torsion measurement. MR images of the proximal femur (**a**) and distal tibia (**b**) with a high grade of artifact degradation. Computed segmentation outlines and reference lines are overlaid. Color coding and image overlays as in Fig. 1. The quality of the segmentation outlines computed by the baseline model (**a**_**1**_ and **b**_**1**_) was too poor for the model to determine any reference lines. In contrast, the models enhanced with default (**a**_**2**_ and **b**_**2**_) and MRI-specific (**a**_**3**_ and **b**_**3**_) augmentation produced segmentation outlines of sufficient quality for determining reference lines. There were no instances in the test data where the model failed to determine the knee reference lines

Automatic angle quantification had variable success rates (Table 3). In this context, “success” is defined as the termination of the model without error and computation of a result. For femoral torsion, the success rate was excellent following default and MRI-specific augmentations, regardless of artifact severity. MRI-specific augmentation delivered measurements in all instances, while default augmentation failed in one. For tibial torsion, the baseline model had substantially lower success rates, particularly with moderate and severe artifacts. For femoral torsion, however, the baseline model delivered largely successful measurements, even with severe artifacts. Exemplary images representing instances where the model failed are presented in Fig. 6.

**Table 3:** Success rates in quantifying femoral and tibial torsion as a function of artifact severity and augmentation strategy

Artifact severity	Augmentation strategy	Femoral torsion	Tibial torsion
Reference	Baseline	100% (20/20)	80% (16/20)
	Default	100% (20/20)	100% (20/20)
	MRI-specific	100% (20/20)	100% (20/20)
Mild	Baseline	100% (6/6)	65% (11/17)
	Default	100% (6/6)	100% (17/17)
	MRI-specific	100% (6/6)	100% (17/17)
Moderate	Baseline	100% (47/47)	58% (18/31)
	Default	100% (47/47)	100% (31/31)
	MRI-specific	100% (47/47)	100% (31/31)
Severe	Baseline	89% (24/27)	3% (1/32)
	Default	96% (26/27)	88% (28/32)
	MRI-specific	100% (27/27)	94% (30/32)

The table provides relative values and absolute counts of completed torsional measurements. If artifact severity of the proximal and distal bone were rated differently, the more severe category would be used to categorize the entire bone

Accuracy was similarly affected by artifact severity and augmentation strategy (Table 4), with post hoc details provided in Supplementary Tables 9–12. The baseline model yielded substantially higher torsional angles across all conditions than default and MRI-specific augmentations and manual measurements. These differences were significant for femoral torsion (left) with moderate and severe artifacts and tibial torsion (right) with mild and moderate artifacts (*p* < 0.001 each). Notably, torsional angles determined based on default or MRI-specific augmentations did not significantly differ from each other or manual measurements. When considering radiologists’ inter-reader means, MAD between manual and automatic measurements were 10.7°, 5.0°, and 4.0° (baseline model, default, and MRI-specific augmentations) for femoral torsion and 19.1°, 4.3° and 3.7° for tibial torsion. ICCs between manual and automatic measurements (each vs. manual measurements) were 0.28 (*p* = 0.11; 95% CI: −0.17 to 0.63 [baseline]), 0.73 (*p* < 0.001; 0.45 to 0.88 [default]), and 0.88 (*p* < 0.001; 0.73 to 0.95 [MRI-specific]) for femoral torsion, and 0.03 (*p* = 0.45; −0.41 to 0.46 [baseline]), 0.92 (*p* < 0.001; 0.81 to 0.97 [default]), and 0.95 (*p* < 0.001; 0.87 to 0.98 [MRI-specific]) for tibial torsion. Similarly, Pearson’s r values between automatic and manual measurements were 0.58 (*p* < 0.001; 0.44 to 0.69 [baseline]), 0.68 (*p* < 0.001; 0.56 to 0.77 [default]), and 0.86 (*p* < 0.001; 0.81 to 0.9 [MRI-specific]) for femoral torsion, and 0.06 (*p* = 0.64; −0.19 to 0.3 [baseline]), 0.80 (*p* < 0.001; 0.73 to 0.86 [default]), and 0.83 (*p* < 0.001; 0.76 to 0.88 [MRI-specific]) for tibial torsion.

**Table 4:** Femoral and tibial torsional angles as a function of artifact severity, measurement method, augmentation strategy, and laterality

Artifact severity	Augmentation strategy	Femur right	Femur left	Tibia right	Tibia left
Reference	Baseline	11.8 ± 10.3	13.3 ± 8.1	48.1 ± 27.8	35.3 ± 28.6
	Default	8.8 ± 6.7	11.0 ± 6.9	37.2 ± 8.0	33.3 ± 7.2
	MRI-specific	8.6 ± 6.8	10.3 ± 7.3	37.4 ± 8.1	33.3 ± 7.4
	Radiologist #1	6.5 ± 7.7	8.2 ± 7.8	38.9 ± 8.9	37.5 ± 6.6
	Radiologist #2	5.6 ± 7.7	8.5 ± 7.0	38.1 ± 9.4	36.5 ± 6.7
	*p*-value	ns	ns	ns	ns
Mild	Baseline	22.5 ± 17.9	25.3 ± 17.5	54.6 ± 26.6	37.7 ± 15.1
	Default	10.7 ± 5.8	17.4 ± 6.8	32.7 ± 5.5	29.2 ± 4.6
	MRI-specific	11.4 ± 7.2	17.0 ± 7.0	33.3 ± 6.3	28.9 ± 4.7
	Radiologist #1	7.1 ± 7.3	12.7 ± 7.1	34.9 ± 7.3	29.9 ± 17.5
	Radiologist #2	5.9 ± 6.5	11.2 ± 7.4	36.6 ± 7.1	33.1 ± 7.0
	*p*-value	ns	ns	***	ns
Moderate	Baseline	10.9 ± 9.3	16.4 ± 13.6	56.3 ± 27.5	38.8 ± 17.6
	Default	9.3 ± 8.5	10.0 ± 11.7	42.1 ± 9.6	36.6 ± 8.2
	MRI-specific	8.7 ± 7.2	10.7 ± 6.2	42.0 ± 10.0	37.4 ± 6.9
	Radiologist #1	6.4 ± 7.8	9.1 ± 8.0	41.0 ± 12.9	40.3 ± 6.5
	Radiologist #2	3.9 ± 7.9	7.1 ± 7.8	42.5 ± 8.1	40.1 ± 6.7
	*p*-value	ns	***	***	ns
Severe	Baseline	13.2 ± 33.0	25.3 ± 24.2	54.3	52.5
	Default	9.3 ± 19.2	12.8 ± 9.5	38.8 ± 9.8	35.5 ± 14.3
	MRI-specific	10.3 ± 16.3	10.0 ± 6.8	39.6 ± 10.0	33.7 ± 7.9
	Radiologist #1	7.3 ± 6.7	7.3 ± 8.2	38.0 ± 10.6	36.2 ± 6.9
	Radiologist #2	4.5 ± 7.8	5.8 ± 8.1	39.4 ± 9.0	35.5 ± 6.7
	*p*-value	ns	***	ns	ns

Torsional angles were compared for specific anatomic regions, sides, and levels of artifact severity between automatic measurements (i.e., different augmentation strategies) and manual measurements (i.e., radiologists #1 and #2) using one-way ANOVA. The corresponding *p*-values are indicated below. *p*-value coding: [***], *p* < 0.001; [ns], non-significant. Data are means ± standard deviation [°]. If no standard deviation is indicated, only one measurement was available. Details of post hoc testing can be found in Supplementary Tables 9–12

## Discussion

This study evaluated the impact of different data augmentation strategies on an AI model’s performance in segmenting and quantifying lower limb alignment, with and without motion artifacts. Using three augmentation strategies, we trained an nnU-Net-based segmentation model and assessed segmentation accuracy and torsional alignment measurements. Our findings indicate that default and MRI-specific augmentations significantly improved model performance compared to no augmentation, particularly in images with moderate or severe artifacts. However, MRI-specific augmentation only marginally (and non-significantly) outperformed default augmentation in severe artifacts. This finding aligns with literature reports indicating that basic augmentation techniques such as flipping, rotating, and transformations significantly enhance diagnostic performance [30, 31]. For automatic whole-prostate segmentation on MRI, Zhang et al investigated the variation in model performance on different external test sets following training with single augmentations. In their study, individual basic augmentations improved model performance by up to 47%, with contrast, brightness, sharpening, and scaling being the most efficient in boosting model performance [32]. Yet, generic one-size-fits-all approaches to data augmentation may not be appropriate. Based on hematologic morphology recognition, Nozaka et al reported that the augmentation strategy needs to be tailored to the specific imaging task to be effective [33].

Surprisingly, domain-specific augmentations in MRI (random blurring, spiking, signal intensity variations, structure rotation/translation, ghosting [25]) only slightly improved over default augmentations. Segmentation of the proximal femur and the distal tibia slightly, yet non-significantly, benefited from MRI-specific augmentation in the presence of severe artifacts. In our study, motion artifacts were induced by repetitive gluteal muscle contraction/relaxation and foot flexion/extension, predisposing the hips and feet to more severe artifacts and enhancing the effectiveness of MRI-specific augmentation. Moreover, the DSC is sensitive to segmented volume size and tends to yield higher values with increasing volumes. This sensitivity may explain higher DSC values for the proximal femur. Nonetheless, this benefit is questionable as visual image quality checks by the radiology technologist during image acquisition may prompt re-acquisition of the sequence or study abortion. Prospective motion correction techniques like PROPELLER, BLADE, and MULTIVANE are widely used clinically to mitigate motion artifacts during image acquisition by compensating for patient motion in real time. These sequence variants have limitations, such as longer scan times and incompatibility with certain imaging protocols or patient populations. Focusing on post-acquisition strategies, our study assessed the value of robust data augmentation during AI model training to enhance segmentation performance when motion artifacts are present despite utilizing prospective motion correction techniques or when such techniques are not feasible. Therefore, data augmentation strategies serve as a valuable “second line of defense” against motion artifacts, complementing prospective techniques by improving image analysis capabilities, salvaging otherwise suboptimal studies, and reducing the need for re-scans.

Despite these marginal improvements, MRI-specific augmentation can provide an additional boost in model performance compared to an elaborate but generalized augmentation approach like the nnU-Net. Its inherent augmentations include rotation, rescaling, and brightness/contrast changes, and it seems sufficient to handle all but the most extreme artifacts. While not specifically tailored and still effective across various imaging modalities such as CT, MRI, and ultrasound, nnU-Net’s generic augmentations may not fully address severe artifacts.

Software packages for in silico data augmentation are widely available (e.g., the batchgenerators, Torchio, and Rising Python libraries [25, 34, 35]) and cover a wide spectrum of standard and specialized image transforms. While we found the artifacts simulated by the augmentation methods employed in our study to emulate real-world artifacts, alternative approaches like generative adversarial networks might be utilized to generate synthetic training images and develop specifically tailored augmentations. However, these approaches are challenging because of high computational demands, the need for large training datasets, and complexities in simulating specific artifacts such as motion artifacts.

The literature on domain-specific versus conventional augmentations is limited but suggests potential benefits from incorporating domain knowledge. For example, training on motion-corrupted data improved the performance of lesion segmentation on brain MRI [36], and myocardial infarction-specific data augmentation improved segmentation accuracy in cardiac MRI studies [37]. Similar to our results, Arega et al noted only marginal improvements, though without statistical validation [38]. In contrast, generating additional training samples based on a shape model did not enhance bone and cartilage segmentation in MRI, underscoring the competitive performance of standard augmentations [39].

Accurate segmentation is essential for accurate quantification of lower limb torsion. Consequently, it is plausible that data augmentation enhanced the model’s robustness (i.e., rate of successful quantifications) and precision (i.e., deviation from manual measurements). In line with the findings above, MRI-specific augmentation provided only marginal improvements over default augmentation in severe artifacts, and the measurements of both augmentations closely matched manual measurements. In contrast, the baseline model significantly overestimated torsional angles.

Our study has limitations. First, our training/validation dataset (*n* = 93 patients) and test dataset (*n* = 20 participants) are relatively small. However, recent studies suggest that even smaller sample sizes of 10–15 are sufficient for bone segmentation using MRI and nnU-Net [39]. Second, we included only data from a single institution, which does not capture the variability in scanners, protocols, sequences, and post-processing routines. However, the nature of this study necessitated a tailored dataset to systematically evaluate the different augmentation strategies on images with varying artifact severity. Nevertheless, our findings lack external validation using datasets from different institutions and imaging setups. True validation is only possible on actual clinical datasets with naturally occurring motion artifacts. Achieving this level of evidence is challenging in a controlled experimental setting; therefore, any augmentation strategy’s robustness remains to be confirmed in real-world settings. Third, our standardized artifact induction method via gluteal and foot motion may not fully represent the less directional motion artifacts encountered in the clinic. By using healthy volunteers and standardized motion induction protocols, we precisely manipulated the type and severity of motion artifacts to ensure consistency. However, the standardization came at the cost of a weaker resemblance to naturally occurring motion artifacts seen in clinical practice. Despite our efforts, motion artifact severity was variable nonetheless, affecting some joints more than others. Overall, our induction method helped realize “standardized variability”. Fourth, we focused only on MRI- and motion-specific artifacts, but other artifacts such as metal, chemical shift, Gibbs, field inhomogeneity, or aliasing artifacts were not considered in this study. Fifth, while the data augmentation strategies effectively improved bone segmentation for quantitative assessment of lower limb alignment, it is important to realize that the AI model does not assess intra-articular pathologies. The imaging protocol is optimized for bone visualization at the hip, knee, and ankle levels for subsequent lower limb alignment quantification. Consequently, its application is limited to these clinical situations, which are often challenging because of motion artifacts. Sixth, the in silico data augmentation may not capture the complex nature of all possible artifacts arising from various patient motions and scanner settings in clinical practice. Simulated motion-induced artifacts only partially resembled real-world artifacts, leading to reduced through-plane correspondence between in silico-generated and real MR image datasets. Still, these augmentations served as effective proxies to help the model learn to recognize anatomy despite motion artifacts. Lastly, the segmentation model was trained on images from pediatric patients but tested on images from adult study participants.

In conclusion, motion artifacts negatively impact the segmentation accuracy and torsional alignment quantification of a diagnostic AI model. Comprehensive general-purpose augmentation strategies, as implemented in nnU-Net, effectively enhance robustness and mitigate motion artifacts. While domain-specific augmentations may provide slight additional performance benefits, models can generally achieve substantial robustness against motion artifacts by utilizing default augmentation configurations. Therefore, we recommend training models using these default comprehensive strategies for most applications, reserving domain-specific augmentations for situations where specialized expertise is available or specific data challenges necessitate a tailored approach.

All procedures performed in studies involving human participants were in accordance with the ethical standards of the institutional and/or national research committee and with the 1964 Helsinki Declaration and its later amendments or comparable ethical standards.

## Supplementary information

ELECTRONIC SUPPLEMENTARY MATERIAL

## Abbreviations

AI: Artificial intelligence
DSC: Dice Similarity Coefficient
ICC: Intraclass correlation coefficient
