# Supplementary material for: AI in motion: the impact of data augmentation strategies on mitigating MRI motion artifacts
Source: Eur Radiol. 2025 May 17;35(11):6865–78. doi: 10.1007/s00330-025-11670-6 (PMC12559149; doi:10.1007/s00330-025-11670-6)
Supplement: Supplementary file 1 — ELECTRONIC SUPPLEMENTARY MATERIAL [file 330_2025_11670_MOESM1_ESM.pdf]

AI In Motion: The Impact of Data Augmentation Strategies on Mitigating MRI Motion Artifacts

ELECTRONIC SUPPLEMENTARY MATERIAL

Supplementary Text 1 - MRI Data Acquisition

For the test dataset, we acquired T2-weighted 2D turbo-spin echo sequences in the axial orientation of 20 participants, covering three stacks over the hips, knees, and ankles that consisted of 29 images each. The following scan parameters were used: field of view 370 *mm*, acquisition matrix 400 x 374 pixels, in-plane resolution (acquired) 0.93 × 0.99 mm/pixel, in-plane resolution (reconstructed) 0.7 × 0.7 mm/pixel, slice thickness 4 *mm*, slice gap 0 *mm*, number of stacks 3, images per stack 29, repetition time 3,985 ms, echo time 80 ms, turbo factor 31, flip angle 90°, number of signal averages 1, scan time (per sequence): 5min 51s.

Supplementary Tables

Supplementary Table 1: Parameters for Augmentation Transforms

[X, Y] denotes the range for which the parameter is randomly sampled so that the parameter may be any value between X and Y. p=X denotes the probability of the transformation being applied. (*) “Default” includes the standard augmentations implemented in the standard nnU-Net (3), such as random rotations, scaling, mirroring, or contrast transforms. In addition, (†) “MRI-specific” details the additional transforms provided by the Torchio python library (4), such as random motion or ghosting transforms.

Augmentation Strategy	Transform	Transform parameters
**Default (*)**	Rotation transform	angle= [-30°, 30°] per axis
	Scale transform	scale factor= [0.7, 1.4]
	Cropping transform	determined dynamically
	Mirror transform	p=0.5 per axis
	Gaussian noise transform	p=0.1, noise variance= [0, 0.1]
	Brightness transform	p=0.15, multiplier range= [0.75, 1.25]
	Contrast transform	p=0.15, contrast range= [0.75, 1.25]
	Downsample transform	p=0.25, zoom range= [0.5, 1]
	Gamma transform	p=0.1, gamma range= [0.7, 1.5]
**MRI-specific (†)**	Blur transform	p=0.5, standard deviation range= [0, 0.8]
	Spike transform	p=0.02, spike number= [0, 3], intensity range= [0, 1.5]
	Bias field transform	p=0.5, coefficient range= [0, 0.5]
	Motion transform	p=0.5, degree range= [-4°, 4°], translation range= [-8mm, 8mm]
	Ghosting transform	p=0.5, ghost number= [0, 5], intensity= [0, 0.5]

Supplementary Table 2: Definitions of Femoral and Tibial Reference Lines

Reference Line	Slice Selection	Method Description	Reference
**Proximal Femur**	Most proximal slice visualizing the femoral head center, femoral neck, and cephalic junction of greater trochanter.	Line connecting the femoral head center (determined by fitting a sphere to the contour of the proximomedial femur segmentation) and the center of the femoral neck (determined by a least-squares fit between the ventral and dorsal femoral neck).	Lee et al. (1)
**Distal Femur**	Slice with the largest area of segmented distal femur.	Line connecting the medial and lateral femoral condyles.	Lee et al. (1)
**Proximal Tibia**	Slice with the largest area of segmented proximal tibia.	Line connecting the medial and lateral tibial condyles.	Strecker et al. (2)
**Distal Tibia**	Slice with the largest width of segmented distal tibia.	Line connecting the centroids of the distal tibia and fibula.	Strecker et al. (2)

Supplementary Table 3: Descriptive Statistics of Artifact Severity

This table presents the distribution of artifact severity across the 300 MR image stacks in the test set. Each stack was evaluated for the presence and extent of motion-induced artifacts, categorized as none, mild, moderate, or severe. The counts and corresponding percentages of each category are presented.

Artifact Severity	Count [n]	Percentage [%]
**None**	69	23
**Mild**	75	25
**Moderate**	96	32
**Severe**	60	20
**Total**	**300**	**100**

Supplementary Table 4: Post-hoc Details of Pair-wise Comparisons of Dice Similarity Coefficients as a Function of Artifact Severity - Proximal Femur

P-values are provided for pair-wise comparisons of Dice Similarity Coefficients based on Tukey's honest significance test. Rows indicate artifact severity and model augmentation. For example, for the segmentation of the proximal femur on reference images, post-hoc testing revealed that the difference between the baseline and default augmentations was not significant (p = .52). Significant differences are indicated in **bold type**.

		Augmentation Strategy		
**Artifact Severity**	**Augmentation Strategy**	**Baseline**	**Default**	**MRI-Specific**
**Reference**	**Baseline**	-	-	-
	**Default**	.52	-	-
	**MRI**	.55	.99	-
**Mild**	**Baseline**	-	-	-
	**Default**	.56	-	-
	**MRI**	.58	.99	-
**Moderate**	**Baseline**	-	-	-
	**Default**	.40	-	-
	**MRI**	.40	>.99	-
**Severe**	**Baseline**	-	-	-
	**Default**	**.002**	-	-
	**MRI**	**<.001**	.18	-

Supplementary Table 5: Post-hoc Details of Pair-wise Comparisons of Dice Similarity Coefficients as a Function of Artifact Severity – Distal Femur

Table details and organization as in **Supplementary Table 4**.

		Augmentation Strategy		
**Artifact Severity**	**Augmentation Strategy**	**Baseline**	**Default**	**MRI-Specific**
**Reference**	**Baseline**	-	-	-
	**Default**	**<.001**	-	-
	**MRI**	**<.001**	.99	-
**Mild**	**Baseline**	-	-	-
	**Default**	**<.001**	-	-
	**MRI**	**<.001**	.99	-
**Moderate**	**Baseline**	-	-	-
	**Default**	**<.001**	-	-
	**MRI**	**<.001**	.99	-
**Severe**	**Baseline**	-	-	-
	**Default**	**.01**	-	-
	**MRI**	**.01**	.99	-

Supplementary Table 6: Post-hoc Details of Pair-wise Comparisons of Dice Similarity Coefficients as a Function of Artifact Severity – Proximal Tibia

Table details and organization as in **Supplementary Table 4**.

		Augmentation Strategy		
**Artifact Severity**	**Augmentation Strategy**	**Baseline**	**Default**	**MRI**
**Reference**	**Baseline**	-	-	-
	**Default**	**<.001**	-	-
	**MRI**	**<.001**	.99	-
**Mild**	**Baseline**	**-**	-	-
	**Default**	**<.001**	-	-
	**MRI**	**<.001**	>.99	-
**Moderate**	**Baseline**	**-**	-	-
	**Default**	**<.001**	-	-
	**MRI**	**<.001**	.99	-
**Severe**	**Baseline**	**-**	-	-
	**Default**	**<.001**	-	-
	**MRI**	**<.001**	.99	-

Supplementary Table 7: Post-hoc Details of Pair-wise Comparisons of Dice Similarity Coefficients as a Function of Artifact Severity – Distal Tibia

Table details and organization as in **Supplementary Table 4**.

		Augmentation Strategy		
**Artifact Severity**	**Augmentation Strategy**	**Baseline**	**Default**	**MRI**
**Reference**	**Baseline**	-	-	-
	**Default**	**<.001**	-	-
	**MRI**	**<.001**	.99	-
**Mild**	**Baseline**	-	-	-
	**Default**	**<.001**	-	-
	**MRI**	**<.001**	>.99	-
**Moderate**	**Baseline**	-	-	-
	**Default**	**<.001**	-	-
	**MRI**	**<.001**	>.99	-
**Severe**	**Baseline**	-	-	-
	**Default**	**<.001**	-	-
	**MRI**	**<.001**	.45	-

Supplementary Table 8: Post-hoc Details of Pair-wise Comparisons of Dice Similarity Coefficients as a Function of Artifact Severity – Distal Fibula

Table details and organization as in **Supplementary Table 4**.

		Augmentation Strategy		
**Artifact Severity**	**Augmentation Strategy**	**Baseline**	**Default**	**MRI**
**Reference**	**Baseline**	-	-	-
	**Default**	**.001**	-	-
	**MRI**	**.002**	.99	-
**Mild**	**Baseline**	**-**	-	-
	**Default**	**.02**	-	-
	**MRI**	**.02**	.99	-
**Moderate**	**Baseline**	**-**	-	-
	**Default**	**<.001**	-	-
	**MRI**	**<.001**	.98	-
**Severe**	**Baseline**	**-**	-	-
	**Default**	**<.001**	-	-
	**MRI**	**<.001**	.92	-

Supplementary Table 9: Post-hoc Details of Pair-wise Comparisons of Femoral Torsion as a Function of Artifact Severity – Femur (Right).

The table provides p-values for pair-wise comparisons of femoral torsional angles based on Tukey's honest significance test. Rows indicate artifact severity and augmentation strategy or radiologist (R1, R2). No significant differences were found.

Artifact Severity	Augmentation Strategy	Baseline	Default	Augmentation Strategy MRI-Specific	R1	R2
Reference	Baseline	-	-	-	-	-
	Default	>.99	-	-	-	-
	MRI-Specific	>.99	>.99	-	-	-
	R1	>.99	>.99	>.99	-	-
	R2	>.99	>.99	>.99	>.99	-
Mild	Baseline	-	-	-	-	-
	Default	>.99	-	-	-	-
	MRI-Specific	>.99	>.99	-	-	-
	R1	>.99	>.99	>.99	-	-
	R2	.98	>.99	>.99	>.99	-
Moderate	Baseline	-	-	-	-	-
	Default	>.99	-	-	-	-
	MRI-Specific	>.99	>.99	-	-	-
	R1	>.99	>.99	>.99	-	-
	R2	.65	>.99	>.99	>.99	-
Severe	Baseline	-	-	-	-	-
	Default	>.99	-	-	-	-
	MRI-Specific	>.99	>.99	-	-	-
	R1	>.99	>.99	>.99	-	-
	R2	.94	>.99	>.99	>.99	-

Supplementary Table 10: Post-hoc Details of Pair-wise Comparisons of Femoral Torsion as a Function of Artifact Severity – Femur (Left).

Table details and organization as in **Supplementary Table 9**. Significant differences are indicated in **bold type**.

Artifact Severity	Augmentation Strategy	Baseline	Default	Augmentation Strategy MRI-Specific	R1	R2
**Reference**	**Baseline**	-	-	-	-	-
	**Default**	>.99	-	-	-	-
	**MRI-Specific**	>.99	>.99	-	-	-
	**R1**	>.99	>.99	>.99	-	-
	**R2**	>.99	>.99	>.99	>.99	-
**Mild**	**Baseline**	-	-	-	-	-
	**Default**	>.99	-	-	-	-
	**MRI-Specific**	>.99	>.99	-	-	-
	**R1**	>.99	>.99	>.99	-	-
	**R2**	>.99	>.99	>.99	>.99	-
**Moderate**	**Baseline**	-	-	-	-	-
	**Default**	.89	-	-	-	-
	**MRI-Specific**	.99	>.99	-	-	-
	**R1**	.47	>.99	>.99	-	-
	**R2**	**.01**	>.99	>.99	>.99	-
**Severe**	**Baseline**	-	-	-	-	-
	**Default**	**.02**	-	-	-	-
	**MRI-Specific**	**<.001**	>.99	-	-	-
	**R1**	**<.001**	>.99	>.99	-	-
	**R2**	**<.001**	>.99	>.99	>.99	-

Supplementary Table 11: Post-hoc Details of Pair-wise Comparisons of Tibial Torsion as a Function of Artifact Severity – Tibia (Right).

Table details and organization as in **Supplementary Table 9**. Significant differences are indicated in **bold type**.

Artifact Severity	Augmentation Strategy	Baseline	Default	Augmentation Strategy MRI-Specific	R1	R2
**Reference**	**Baseline**	**-**	**-**	**-**	**-**	**-**
	**Default**	.88	**-**	**-**	**-**	**-**
	**MRI-Specific**	.92	>.99	**-**	**-**	**-**
	**R1**	>.99	>.99	>.99	**-**	**-**
	**R2**	.98	>.99	>.99	>.99	**-**
**Mild**	**Baseline**	**-**	**-**	**-**	**-**	**-**
	**Default**	**<.001**	**-**	**-**	**-**	**-**
	**MRI-Specific**	**<.001**	>.99	**-**	**-**	**-**
	**R1**	**.002**	>.99	>.99	**-**	**-**
	**R2**	**.02**	>.99	>.99	>.99	**-**
**Moderate**	**Baseline**	**-**	**-**	**-**	**-**	**-**
	**Default**	**.04**	**-**	**-**	**-**	**-**
	**MRI-Specific**	**.04**	>.99	**-**	**-**	**-**
	**R1**	**.007**	>.99	>.99	**-**	**-**
	**R2**	.07	>.99	>.99	>.99	**-**
**Severe**	**Baseline**	**-**	**-**	**-**	**-**	**-**
	**Default**	>.99	**-**	**-**	**-**	**-**
	**MRI-Specific**	>.99	>.99	**-**	**-**	**-**
	**R1**	>.99	>.99	>.99	**-**	**-**
	**R2**	>.99	>.99	>.99	>.99	**-**

Supplementary Table 12: Post-hoc Details of Pair-wise Comparisons of Tibial Torsion as a Function of Artifact Severity – Tibia (Left).

Table details and organization as in **Supplementary Table 9**.

Artifact Severity	Augmentation Strategy	Baseline	Default	Augmentation Strategy MRI-Specific	R1	R2
Reference	Baseline	-	-	-	-	-
	Default	>.99	-	-	-	-
	MRI-Specific	>.99	>.99	-	-	-
	R1	>.99	>.99	>.99	-	-
	R2	>.99	>.99	>.99	>.99	-
Mild	Baseline	-	-	-	-	-
	Default	>.99	-	-	-	-
	MRI-Specific	>.99	>.99	-	-	-
	R1	>.99	>.99	>.99	-	-
	R2	>.99	>.99	>.99	>.99	-
Moderate	Baseline	-	-	-	-	-
	Default	>.99	-	-	-	-
	MRI-Specific	>.99	>.99	-	-	-
	R1	>.99	>.99	>.99	-	-
	R2	>.99	>.99	>.99	>.99	-
Severe	Baseline	-	-	-	-	-
	Default	>.99	-	-	-	-
	MRI-Specific	>.99	>.99	-	-	-
	R1	>.99	>.99	>.99	-	-
	R2	>.99	>.99	>.99	>.99	-

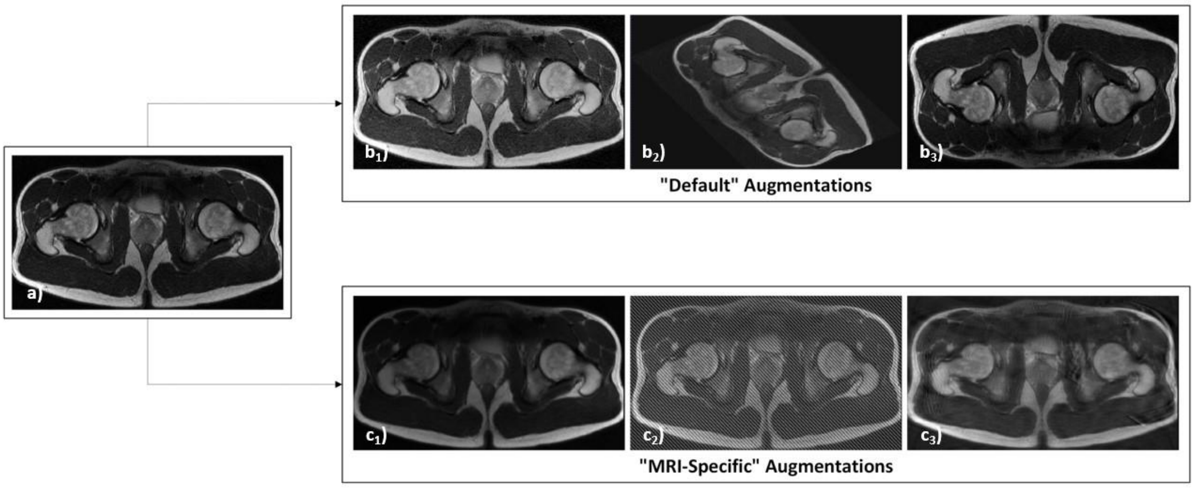

Supplementary Figure 1: Example Images of Augmentations

Representative images of the proximal femur with different image transforms are shown. a) Original (unaugmented) MR image. b) Default augmentations as implemented in the nnU-net architecture. b_1) Intensity adjustments of contrast, gamma, and additional Gaussian noise. $b_{2,3}$) Spatial augmentations, i.e., rotation (b_2) and mirroring (b_3). c) MRI-specific augmentations. c_1) Random blur and bias fields. c_2) Spiking artifacts. c_3) Random motion and ghosting.

References

1. Lee YS, Oh SH, Seon JK, Song EK, Yoon TR. 3D femoral neck anteversion measurements based on the posterior femoral plane in ORTHODOC® system. Medical and Biological Engineering and Computing. 2006;44:895–906.

2. Strecker W, Keppler P, Kinzl L. Posttraumatische Beindeformitäten. In: Strecker W, Keppler P, Kinzl L, editors. Posttraumatische Beindeformitäten [Internet]. Berlin, Heidelberg: Springer Berlin Heidelberg; 1997 [cited 2024 Aug 22]. p. 3–6. Available from: http://link.springer.com/10.1007/978-3-642-60727-1_1

3. nnU-Net Github Repository [Internet] [cited 2024 Aug 22]. Available from: https://github.com/MIC-DKFZ/nnUNet

4. Pérez-García F, Sparks R, Ourselin S. TorchIO: A Python library for efficient loading, preprocessing, augmentation and patch-based sampling of medical images in deep learning. Computer Methods and Programs in Biomedicine. 2021;208:106236.
